# Discriminating Interference Fading Locations in Φ-OTDR Using Improved Density Clustering Algorithm

**DOI:** 10.3390/s25227084

**Published:** 2025-11-20

**Authors:** Hongyu Tao, Miao Yu, Zhaoyang Zhang, Shijie Li, Huan Liu, Guangxi Li, Mingyang Sun

**Affiliations:** 1 School of Electronic and Information Engineering, Changchun University, Changchun 130000, China; thyu0112@163.com (H.T.); liuh@ccu.edu.cn (H.L.); ligq@ccu.edu.cn (G.L.); 2 Shenzhen Institutes of Advanced Technology, Chinese Academy of Sciences, Shenzhen 518000, China; miao.yu2@siat.ac.cn

**Keywords:** optical fiber sensors, interference fading, adaptive signal processing, AP-DBSCAN, nearest-neighbor interpolation

## Abstract

The phase-sensitive optical time-domain reflectometer (Φ-OTDR) system is a distributed optical fiber sensing technology capable of measuring weak vibration signals in real time. However, while the use of a narrow-linewidth laser source enhances the system’s sensitivity, the accompanying high coherence introduces an inherent drawback: fading noise. This phenomenon can lead to significant phase demodulation distortion, severely compromising the system’s reliability. Consequently, interference fading represents a fundamental challenge in Φ-OTDR systems. We propose an optimized density clustering algorithm, termed adaptive principal component analysis DBSCAN++ (AP-DBSCAN). The procedure begins by identifying fading regions based on the fading principle. Subsequently, AP-DBSCAN integrates the K-distance to adaptively determine parameters, and incorporates PCA technology and the DBSCAN++ algorithm to efficiently and accurately distinguish fading points within these regions. Finally, the compromised data points are reconstructed using a nearest-neighbor interpolation method. Experimental results demonstrate the superior performance of the proposed method over DBSCAN, FDBSCAN, and DBSCAN++. Our approach achieves adaptive determination of the eps and Minpts parameters, maintaining a high fading-point detection accuracy of 99.92% while significantly improving computational efficiency by 67.33% to 76.29%.

## 1. Introduction

Phase-sensitive optical time-domain reflectometry (Φ-OTDR) stands as a cornerstone advancement in distributed optical fiber sensing. The analysis of phase perturbations in the Rayleigh backscattered light within the optical fiber enables high-sensitivity, long-range, and real-time localization and monitoring of vibration events along the sensing cable [1]. Its unique, fully distributed sensing architecture requires only a single fiber to achieve coverage extending over tens of kilometers. This technology also boasts remarkable advantages, including immunity to electromagnetic interference, high concealment, and excellent environmental adaptability [2]. Consequently, Φ-OTDR has been widely deployed in diverse fields such as security early warning systems for oil and gas pipelines [3,4,5], seismic wave detection [6,7,8], perimeter security for border areas [9,10,11], and underwater activity monitoring [12,13].

When external vibrations act upon an optical fiber, they alter the fiber’s refractive index or length, resulting in minute phase shifts in the backscattered Rayleigh light [14]. However, the randomly distributed scattering points in the optical fiber can cause destructive interference of the Rayleigh backscattered light from the coherent detection pulse. This interference may introduce noise into the Φ-OTDR intensity signal, resulting in significant errors during phase demodulation and potentially leading to false alarms [15,16]. Therefore, accurately locating the positions of interference fading points in optical fibers becomes a critical prerequisite for mitigating their adverse effects. Given that fading points manifest as statistical outliers with amplitudes significantly deviating from normal points, outlier detection methods such as the standard deviation approach or Grubbs’ test have been proposed as alternative solutions. However, the standard deviation method [17] is constrained by its assumption of a normal distribution and its sensitivity to multiple outliers, while Grubbs’ test [18] performs poorly in handling large volumes of outliers and involves subjective selection of the significance level.

Given the density characteristics observed in the data points, the application of a density-based clustering algorithm is highly appropriate. This method is capable of identifying high-density areas and marking low-density points as noise or outliers. Commonly used algorithms include DBSCAN, FDBSCAN, and DBSCAN++. Among them, DBSCAN is a foundational algorithm proposed by Ester et al. in 1996. It is designed to discover clusters of arbitrary shapes within spatial or high-dimensional data that contain noise [19]. The DBSCAN algorithm requires two global parameters: the neighborhood radius (eps) and the minimum number of points (MinPts). An object is classified as a core point if there are at least MinPts neighbors within its eps. All objects within the neighborhood of a core point are directly density-reachable and form an initial cluster. This cluster is then expanded by incorporating the neighborhoods of any other core points within it. Objects inside a cluster that do not meet the core point condition are labeled as border points, while any object that is not reachable from any core point is categorized as noise. FDBSCAN eliminates redundant neighborhood evaluations by directly assigning core points to clusters. These clusters may either retain their initial assignments or expand through density-reachable overlaps with adjacent clusters [20]. If a border object is adjacent to a core point, the algorithm assigns it to the cluster containing that core point. Otherwise, it is classified as noise. DBSCAN++ is a straightforward refinement of the original DBSCAN algorithm, which requires density calculations only for a selected subset of points [21]. Its core principle operates on the principle of estimating densities for a subset of *m* data points (where *m* << *n*), rather than for the entire dataset of size *n*. Subsequently, standard DBSCAN operations are performed on this subset. However, these density-based clustering algorithms exhibit low computational efficiency when processing the high-dimensional data acquired by Φ-OTDR systems.

To address the aforementioned challenges, this paper proposes the AP-DBSCAN algorithm, which can autonomously determine its parameters, rapidly process high-dimensional data, and accurately identify the locations of fading points. The work begins with a detailed analysis of distributed optical fiber sensing, interference fading, and the differential principle of fading. Subsequently, the AP-DBSCAN algorithm is applied to tackle the problem of efficient fading point discrimination. To handle the high-dimensional data, principal component analysis (PCA) is employed to reduce dimensionality and extract salient features [22]. The two parameters, eps and MinPts, are determined adaptively. Specifically, the eps value is derived by analyzing the k-distance distribution across the entire dataset [23], whereas the MinPts parameter is determined through an analysis of the density distribution based on the pre-computed eps threshold. Following this, the identification of fading points is performed by applying the DBSCAN++ algorithm to the data. Following the identification of fading points, the corresponding locations in the phase signal are flagged as missing values. For fading suppression, existing methods include suppressing interference fading based on differential phase shift pulse technology [24] and suppressing interference fading by using phase shift transformation [25]. In this paper, the nearest-neighbor interpolation method is utilized. The positions of the fading points of the phase signal are set as missing values, and then the missing points are filled with the nearest non-missing values to eliminate the influence of interference fading. The superiority of the proposed AP-DBSCAN method was first validated through simulation experiments, demonstrating its advantages over DBSCAN, FDBSCAN, and DBSCAN++. Finally, an experimental system was set up to collect actual disturbance signals in order to verify the processing capability of the AP-DBSCAN method for actual signals.

## 2. Fundamental Principles

### 2.1. Principle of Distributed Optical Fiber Sensing

**Figure 1:**
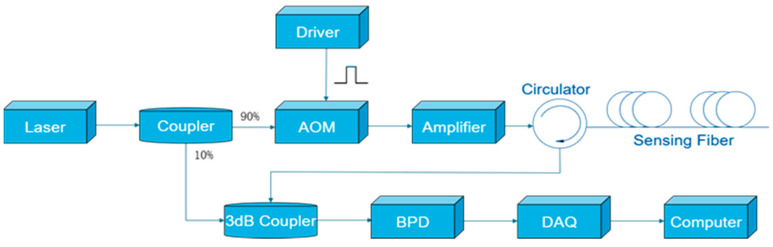
Digital coherent detection scheme for Φ-OTDR.

As illustrated in Figure 1, the system employs a RIO laser as the transmitter. Its output light is split by a fiber optic coupler, with 90% serving as the probe beam and 10% acting as the local reference beam. The probe light is then modulated by an acoustic-optic modulator (AOM) into pulsed light, introducing a frequency shift (Δf). After being amplified by an Erbium-Doped Fiber Amplifier (EDFA), the pulsed light is injected into the sensing fiber. The generated Rayleigh backscattered light returns and is coherently mixed with the local reference beam in a 3 dB coupler. The resulting beat signal is converted into an electrical signal by a balanced photodetector (BPD). Finally, this electrical signal is captured by a high-speed data acquisition (DAQ) card and processed using a built-in I/Q demodulation algorithm to extract the amplitude and phase information distributed along the optical fiber.

The backward Rayleigh scattered light at a position Z along the optical fiber can be expressed as follows [26]:


(1) $$E_{S}(z,t)=r(z,t)E_{0}f(t)\cos\theta \exp\left(-\frac{\alpha z}{2}-j\omega_{s}t\right)\exp\left(j2\int_{0}^{z}\beta dy\right)$$

In the above equation, β is the propagation constant, expressed as $\beta =2\pi n_{f}\left[T(z),\varepsilon (z)\right]/\lambda$; r(z,t) is the random Rayleigh scattering coefficient; and $f\left(\begin{array}{c} t \end{array}\right)$ represents the optical pulse waveform. The term cosθ is the polarization-dependent factor, where θ denotes the angle between the polarization states of the probe light and the local oscillator light. The signal acquired by the digital acquisition card is processed using an I/Q demodulation algorithm to obtain two orthogonal signal components.


(2) $$I\propto E_{s}(z)E_{L}(z)\cos\phi_{s}(z)$$

(3) $$Q\propto E_{S}(z)E_{L}(z)\sin\phi_{s}(z)$$

In the above equation, $E_{S}$ represents the Rayleigh backscattered light, $E_{L}$ represents the local light, and z indicates the position along the optical fiber. The amplitude and phase information are obtained by combining the I and Q signals.

### 2.2. Principle of Interference Fading Differential

Interference fading in optical fibers can typically be understood as the result of the superposition of numerous scattering points of wavelength-scale within the pulse width region. Since both the amplitude and the phase originate from the same down-converted digital signal (I/Q components) at the same sampling instant, the positions exhibiting amplitude fading must necessarily coincide with the positions experiencing phase fading [27]. Given that differentiation quantifies a function’s rate of change, it can be used to pinpoint fading events, which are marked by rapid amplitude attenuation. The amplitude A(z) is described by the following equation [28]:

(4) $$\begin{array}{cc} A(z) & =E_{L}E_{S}r_{c}(z)e^{-\alpha z}\cos(\theta )\\ & =E_{L}E_{S}e^{-\alpha z}\cos(\theta )\int_{\pi }^{z+\Delta z}rf(x/\nu )\exp(j{\left[2(\omega /c)\right]}_{z}^{x}\Delta n_{eff}(y)dy+2n_{eff}\Delta \omega /c]dx \end{array}$$

Fading corresponds to the condition where A(z) is very low or even zero. Consequently, the terms in the equation that do not contribute to the fading of A(z) can be treated as constants. Let: $a=E_{L}(z)E_{0}e^{-\alpha z},b=\bar{r}f(x/v),c=2(\omega /c),d=2{\bar{n}}_{eff}\Delta \omega /c$, and convert the complex function into a real function, obtaining the following:

(5) $$A\left(z\right)\propto \cos\left(\theta \left(z\right)\right)\sqrt{{\left(a\int_{z}^{z+\Delta z}b\cos\left(c\int_{0}^{x}\Delta n_{eff}\left(y\right)dy+d\right)dx\right)}^{2}}$$

When interference fading occurs, by analyzing $\int_{z}^{z+\Delta z}\;F(x)dx$, the remaining terms are treated as constants, the first-order derivative is given as follows:

(6) $$\begin{array}{cc} A^{\prime }(z) & =\frac{d}{dz}(\sqrt{{\left(\int_{z}^{z+\Delta z}b\cos(c\int_{0}^{x}\Delta n_{eff}(y)dy+d)dx\right)}^{2}})\\ & =\frac{\int_{z}^{z+\Delta x}b\cdot \cos(c\int_{0}^{x}\Delta n_{eff}(y)dy+d)dx\cdot \left(b\cdot \cos(c\cdot \int_{0}^{x+\Delta x}\Delta n_{eff}(y)dy+d)-b\cdot \cos(c\cdot \int_{0}^{x}\Delta n_{eff}(y)dy+d)\right)}{\sqrt{{\left(\int_{z}^{z+\Delta x}b\cos(c\int_{0}^{x}\Delta n_{eff}(y)dy+d)dx\right)}^{2}}} \end{array}$$

The amplitude variation of the optical fiber between z and +Δz does not abruptly become zero; interference fading occurs if and only if $bcos(c\int_{0}^{x}\;\Delta n_{eff}(y)dy+d)=0$. When the variation in the refractive index causes $\Delta n_{eff}(y)dy+d$→π/2+kπ the left-hand and right-hand derivatives of $A^{\prime }(z)$ exhibit a discontinuity. Hence, the point at which fading occurs corresponds to a point of non-differentiability.

**Figure 2:**
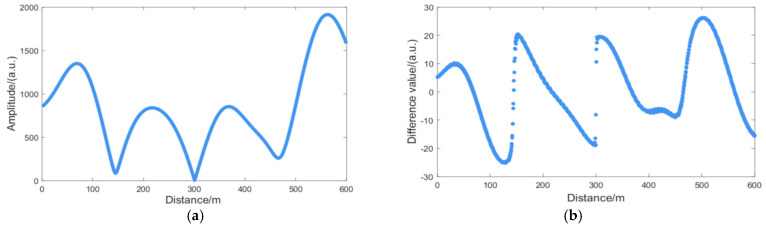
First derivative of the amplitude information: (**a**) amplitude information; (**b**) amplitude derivative once.

Figure 2a presents the amplitude, where the fading points are identified as sharp dips with low magnitude. Subsequently, Figure 2b exhibits a clear discontinuity at these fading points, which is caused by interference fading.

## 3. AP-DBSCAN Principle and Fading Point Discrimination

Based on the characteristics of the data, principal component analysis (PCA) is employed for data processing. PCA performs unsupervised dimensionality reduction through eigendecomposition of the covariance matrix. It derives orthogonal components from eigenvectors of standardized data, ordered by descending eigenvalues. This transforms correlated variables into uncorrelated low-dimensional features with minimal information loss. Applied to Φ-OTDR signals, PCA extracts dominant components through dimensionality reduction. This critical preprocessing step significantly enhances subsequent fading point identification efficiency.

The adaptive determination of eps utilizes the K-distance method, where K = dimensionality + 1. This configuration ensures adaptive K-value selection while avoiding self-distance, thereby preventing local density misjudgment. For each data point, the distances to its K nearest neighbors are computed and sorted in ascending order. The eps value is then defined as a sufficiently high percentile of these sorted K-distances across the entire dataset. Subsequently, with eps determined, each sample point in the dataset is traversed to count the number of points lying within its eps-radius neighborhood. The arithmetic mean of these neighborhood counts across all samples is computed, and its ceiling (upward-rounded integer value) is designated as the MinPts parameter.

**Figure 3:**
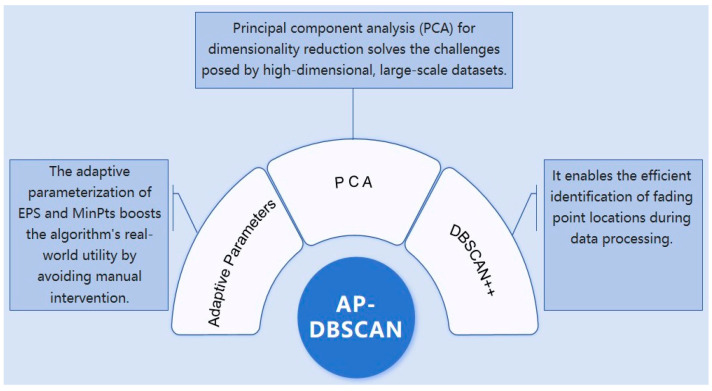
Algorithm structure.

The two adaptively determined parameters are then utilized in conjunction with DBSCAN++. The core principle of DBSCAN++ involves data sampling, for which a uniform sampling strategy is adopted in this study. This approach preserves the original DBSCAN’s robustness to noise and its ability to identify clusters of arbitrary shapes while notably reducing computational complexity, thereby offering significant advantages when processing large-scale, high-dimensional data. A schematic diagram of the algorithm is presented in Figure 3.

**Figure 4:**
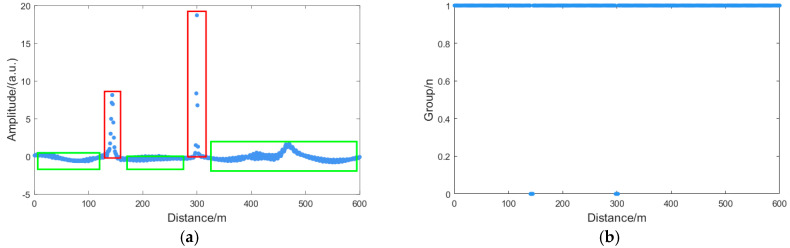
Algorithm processing of the digitally differentiated amplitude information: (**a**) third derivative of the amplitude information; (**b**) discrimination results. The red area indicates the area of decline, while the green area represents the normal area.

**Figure 5:**
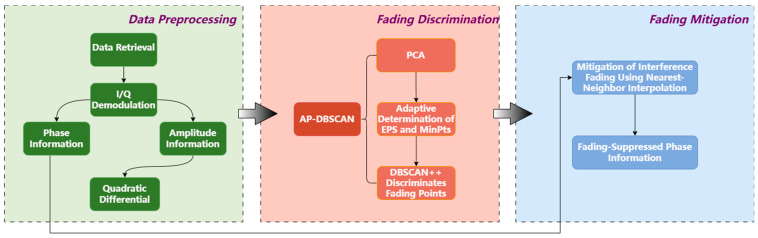
Flowchart of the discrimination process.

In summary, the AP-DBSCAN algorithm integrates PCA for initial data processing. The processed data subsequently undergoes clustering via DBSCAN++ with adaptively determined parameters, enabling rapid identification of fading point locations. Analysis of the differentiated amplitude information, as illustrated in Figure 4a, reveals that normal points exhibit high density and form compact regions, whereas fading points demonstrate low density and sparse distribution. The red areas indicate fading regions, while the green areas represent normal regions. The result of applying the AP-DBSCAN algorithm to process the differentiated amplitude information is shown in Figure 4b, where Cluster 1 corresponds to normal points and other clusters denote fading points. Following the identification of fading points, the nearest-neighbor interpolation method is employed to reconstruct the impaired data at these locations. The overall flowchart of the fading suppression process is presented in Figure 5.

## 4. Experiments

### 4.1. Experiment on Fading Suppression in Vibration Signals

**Figure 6:**
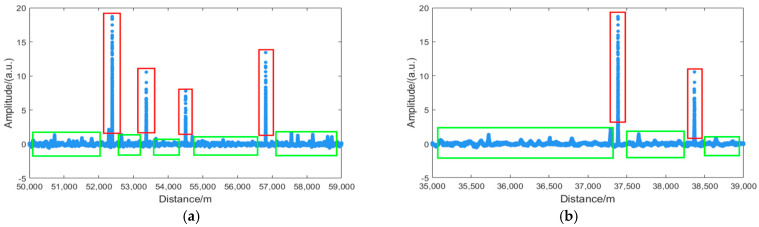
Second derivative of the amplitude: (**a**) Longitudinal amplitude second-order differential; (**b**) Second-order differential of short-distance amplitude. The red area indicates the area of decline, while the green area represents the normal area.

The experiment was conducted on a sensing fiber with a total length of 70,080 m. A PZT vibration at 500 Hz was applied to the fiber segment between 48,610 m and 48,850 m. The type of optical fiber used is G652D 9/125um single-mode optical fiber. The manufacturer is Fiberhome. To validate the algorithm’s capability for both long-range and short-range fading point identification, a segment from 50,000 m to 59,000 m was selected for long-range detection, and a segment from 35,000 m to 39,000 m was chosen for short-range detection, thereby verifying the algorithm’s feasibility and accuracy. As shown in Figure 6a,b, which display the second derivative of the amplitude information for the long-range and short-range sections, respectively, the red areas indicate fading regions and the green areas represent normal regions.

**Figure 7:**
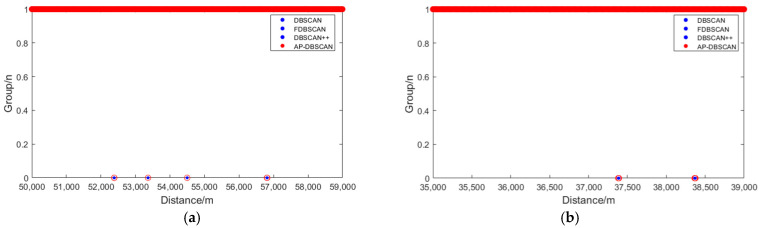
Algorithm scatter plot: (**a**) Long-distance fading location discrimination; (**b**) Short-distance fading location discrimination.

The DBSCAN, FDBSCAN, DBSCAN++, and AP-DBSCAN algorithms were, respectively, applied to cluster the long-range and short-range regions. For DBSCAN, FDBSCAN, and DBSCAN++, the parameters were set as Eps = 5 and MinPts = 5. In contrast, AP-DBSCAN determined its parameters adaptively. Regarding clustering performance, as shown in Figure 7a,b, the solid blue dots represent the fading locations identified by DBSCAN, FDBSCAN, and DBSCAN++, while the hollow red circles denote the fading locations identified by AP-DBSCAN. It can be observed that all four algorithms identified fading points at identical locations and in equal quantities across both the long- and short-range regions, confirming the feasibility of AP-DBSCAN.

In terms of clustering efficiency, Table 1 presents the computational time required by the four clustering algorithms when processing the differentiated data. Compared to the three traditional clustering algorithms, the proposed AP-DBSCAN achieves identical accuracy in identifying fading locations. Among the conventional methods, DBSCAN exhibits the longest processing time while maintaining the highest accuracy in fading point localization. FDBSCAN reduces computational time compared to DBSCAN without compromising accuracy, primarily due to the elimination of redundant operations in overlapping regions of core objects’ neighborhoods. DBSCAN++ demonstrates significant improvement in processing speed over DBSCAN, owing to its data sampling procedure. The proposed AP-DBSCAN algorithm achieves the fastest processing performance by incorporating PCA technology, which substantially enhances computational speed while enabling autonomous parameter determination. This improvement results in a 30.28% to 72.51% increase in clustering efficiency compared to the other methods.

**Figure 8:**
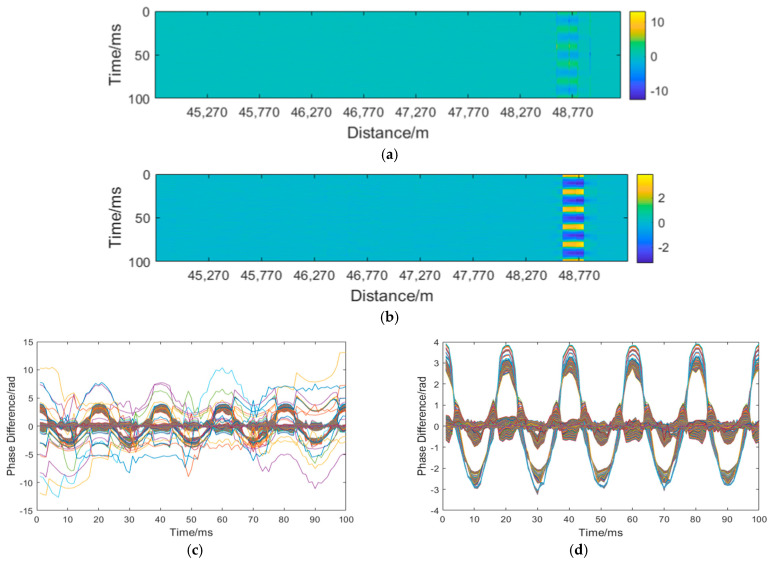
Phase compensation: (**a**) unmodified spacetime domain map; (**b**) Corrected spatiotemporal domain map; (**c**) unmodified signal diagram; (**d**) corrected signal diagram.Different colors represent different dimensions of the curves.

Following the identification of interference fading points, this study employs the nearest-neighbor interpolation method to efficiently reconstruct the impaired data at these locations, enabling rapid and accurate detection and correction of fading effects. As shown in Figure 8a, from 48,610 m to 48,850 m is the vibration zone. Due to the influence of interference fading, the vibration signal is drowned out. Figure 8b displays the reconstructed signal, where the vibration region becomes distinctly visible. Figure 8c shows the phase difference information before correction, exhibiting chaotic phase jumps. After reconstruction, clear signal characteristics can be observed in Figure 8d. Due to the fading effects, the original signal achieves a signal-to-noise ratio (SNR) of only 2.5985 dB prior to correction. Following reconstruction, the SNR improves to 2.6389 dB. The SNR is calculated as follows:

(7) $$N_{dB}=10\mathrm{lg}\frac{P_{s}}{P_{n}}$$

where $P_{s}$ denotes the signal power, and $P_{n}$ represents the noise power.

The amplitude signal is first analyzed through differential operation and AP-DBSCAN clustering to detect the locations of signal fading. If no fading regions are detected, the signal remains unaltered. For identified fading segments, the corresponding regions undergo correction based on neighborhood interpolation. The affected areas will be shielded, and the intervals of this shielding will subsequently be replaced by adjacent non-fading data points. When no adjacent values are available on the left side, the gaps are filled entirely using values from the right side, and vice versa. This process ensures phase continuity at all corrected locations, ultimately yielding a reconstructed phase signal.

### 4.2. Experiment on Fading Suppression in Underwater Acoustic Signals

**Figure 9:**
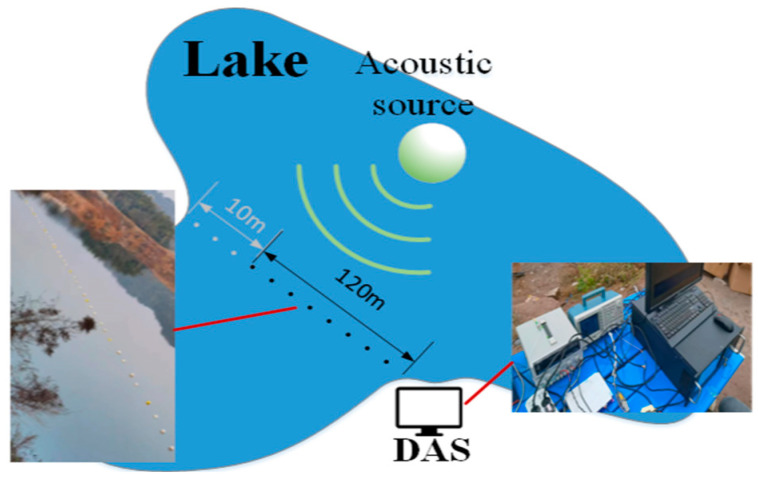
Schematic diagram and experimental setup of underwater acoustic signal fading suppression.

The experiment utilized hydroacoustic signals collected from a lake, with the setup shown in Figure 9. A Φ-OTDR system was shore-deployed with a 120 m coiled optical fiber cable. The optical fibers used in this study are specially customized ones. The cable’s end was anchored across the bank with a 10 m rope, stabilized at 1 m depth using 5 m spaced floats and counterweights. A kayak-mounted UPS-powered acoustic source traversed the cable path, emitting specific frequencies at designated locations.

**Figure 10:**
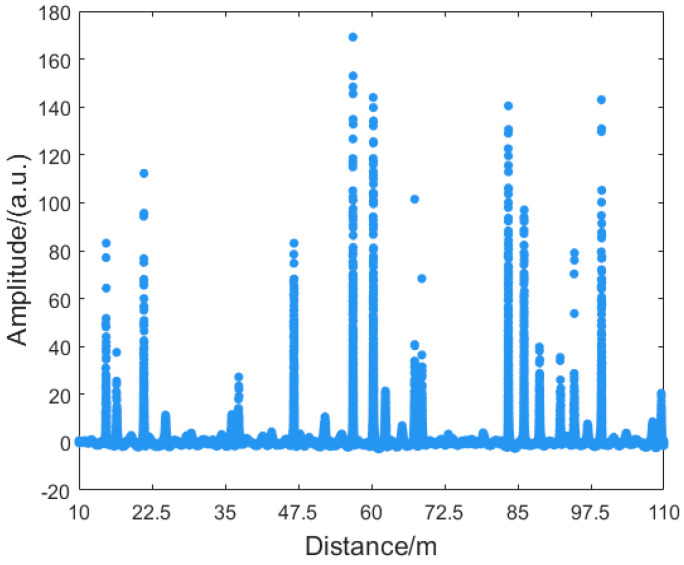
Secondorder derivative of the amplitude signal.

The boat was positioned 50 m perpendicular and 27 m parallel to the cable front. A 36 V/180 Hz acoustic signal was emitted. Based on the fading discrimination framework and the proposed method, the fading point locations were rapidly identified. Interference fading was suppressed using the nearest-neighbor interpolation method. Figure 10 shows the second-order derivative plot of the signal.

**Figure 11:**
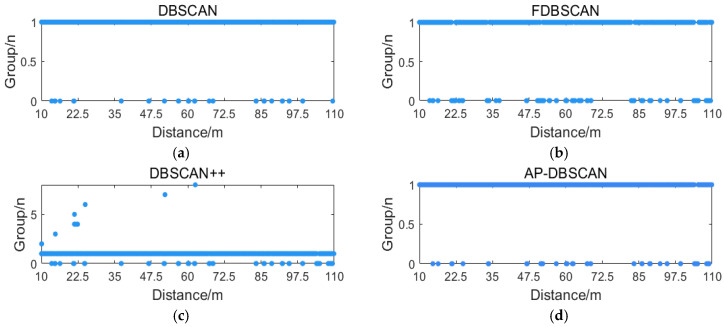
Underwater fading point discrimination: (**a**) DBSCAN scatter plot; (**b**) FBSCAN scatter plot; (**c**) DBSCAN++ scatter plot; (**d**) AP-DBSCAN scatter plot.

For the processed underwater acoustic signal after fading differentiation, DBSCAN, FDBSCAN, DBSCAN++, and AP-DBSCAN were applied to compare their performance in terms of processing time and accuracy for fading point identification. As shown in Figure 11, the fading locations identified by the four algorithms are illustrated.

As illustrated in the figure, DBSCAN achieves relatively accurate identification of fading points in both high- and low-density regions. FDBSCAN exhibits an increased misjudgment rate compared to DBSCAN. This performance degradation stems from its limited capability in handling high-dimensional and large-volume datasets. Although FDBSCAN simplifies computational procedures by verifying whether core objects share labeled neighbors to facilitate cluster merging, this strategy introduces sequential dependencies: erroneous labeling in early stages can propagate inaccuracies, adversely impacting subsequent merging processes. Among the four algorithms, DBSCAN++ demonstrates the highest misjudgment rate. Its core approach involves uniform data sampling, which reduces dataset scale and mitigates redundancy, but introduces critical sensitivity to the eps and MinPts parameters. In particular, inadequate sampling density frequently leads to misclassification.

In comparison, the proposed AP-DBSCAN algorithm achieves fully autonomous parameter determination while maintaining a very low error rate. It adaptively configures eps and MinPts according to variations in data dimensionality and volume, thereby substantially enhancing detection accuracy. Relative to conventional DBSCAN, AP-DBSCAN attains an accuracy of 99.92%.

**Figure 12:**
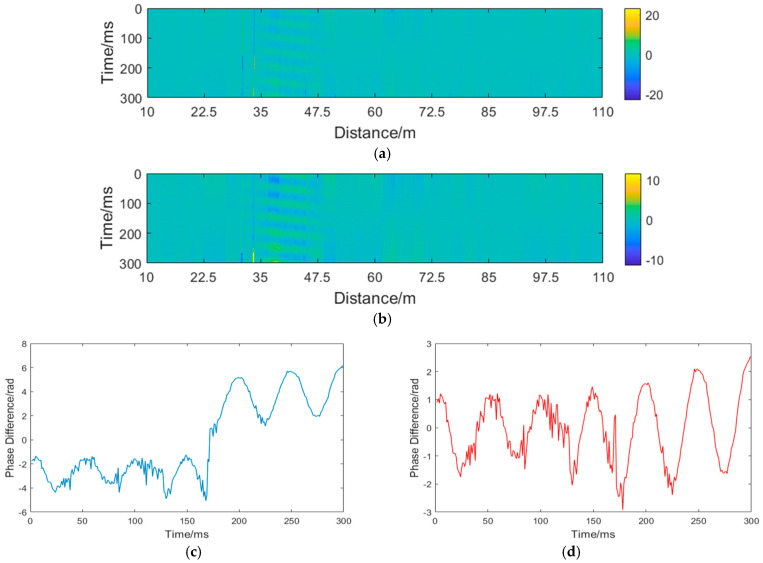
Underwater phase compensation: (**a**) unmodified spacetime domain map; (**b**) Corrected spatiotemporal domain map; (**c**) unmodified signal diagram; (**d**) corrected signal diagram.

As can be observed from Table 2, the processing time required by AP-DBSCAN is significantly shorter than that of the other algorithms. Overall, while maintaining accuracy, AP-DBSCAN demonstrates a substantial advantage in discrimination time, considerably reducing the overall computational overhead. This improvement results in a 67.33% to 76.29% increase in clustering efficiency compared to the other methods. Following the identification of fading points, the nearest-neighbor interpolation method is similarly applied to mitigate the effects of interference-induced fading, as illustrated in Figure 12.

Figure 12a displays the uncorrected signal, where the overall signal is significantly affected by fading. Multiple locations exhibit substantial phase jumps due to fading interference. Moreover, the resulting fading noise has submerged the disturbance signal, with only a vague outline of the disturbance signal discernible in the figure. Figure 12b presents the corrected signal, where most fading points have been eliminated, allowing for clear identification of the disturbance location. Figure 12c shows the phase before correction, revealing severe phase jumps. In Figure 12d, after correction, the phase jumps are normalized, exhibiting a sinusoidal pattern. Due to interference fading, the SNR before correction was 2.1713 dB. After correction, the SNR improved to 3.0647 dB.

## 5. Conclusions

This paper presents an AP-DBSCAN algorithm specifically designed for locating the fading points in φ-OTDR systems. To address the issue of low computational efficiency in processing high-dimensional signals acquired by Φ-OTDR systems, the method incorporates principal component analysis for feature extraction and dimensionality reduction. By integrating the K-distance method into DBSCAN++, it achieves adaptive determination of the eps and MinPts parameters. Simulation experiments demonstrate the superiority of AP-DBSCAN over DBSCAN, FDBSCAN, and DBSCAN++ in terms of temporal efficiency for fading point identification, and its effectiveness is further validated using underwater acoustic signals. Experimental results indicate that AP-DBSCAN achieves the optimal processing time compared to DBSCAN, FDBSCAN, and DBSCAN++, with recorded processing times of 21.163 min, 18.744 min, 15.361 min, and 5.017 min, respectively. This confirms that the proposed method significantly improves the efficiency of fading point discrimination while maintaining an accuracy rate of 99.92%. The interference fading effect on the signal is subsequently eliminated using nearest-neighbor interpolation. AP-DBSCAN successfully resolves the time-consuming challenges associated with processing high-dimensional signals and realizes full parameter adaptivity. This work holds significant importance for the efficient discrimination of fading points, suppression of interference fading, and reconstruction of signals in practical Φ-OTDR systems.

## Figures and Tables

**Table 1:** Comparative results of computational time for the four algorithms.

Algorithm	Long-Range Processing Time	Short-Range Processing Time
DBSCAN	28.739 s	15.633 s
FDBSCAN	20.807 s	10.925 s
DBSCAN++	11.330 s	6.474 s
**AP-DBSCAN**	**7.899 s**	**2.728 s**

**Table 2:** Comparative results of computational time for the four algorithms.

Algorithm	Processing Time Comparison
DBSCAN	21.163 min
FDBSCAN	18.744 min
DBSCAN++	15.361 min
**AP-DBSCAN**	**5.017 min**

## Data Availability

The raw data supporting the conclusions of this article will be made available by the authors on request.
